# General Approach to the Synthesis of Prochiral Atropisomeric Biaryls

**DOI:** 10.5402/2011/919102

**Published:** 2011-06-26

**Authors:** Katarzyna Kielar, Oleg M. Demchuk, K. Michał Pietrusiewicz

**Affiliations:** Department of Organic Chemistry, Maria Curie-Skłodowska University, 33-Gliniana Street, 20-614 Lublin, Poland

## Abstract

General approach to the synthesis of prochiral precursors of chiral atropisomeric biaryls based on several complementary methods has been developed. Biaryls were obtained in good to excellent yields depending on their structure and selected method of synthesis. Furthermore, we demonstrate a possibility of utilisation of the obtained compounds possessing 2 or 3 *ortho* substituents around the aryl-aryl bond in direct and directed arylation reaction leading through transition metal-mediated C–H bond activation to atropisomeric compounds.

## 1. Introduction

**Scheme 1:**
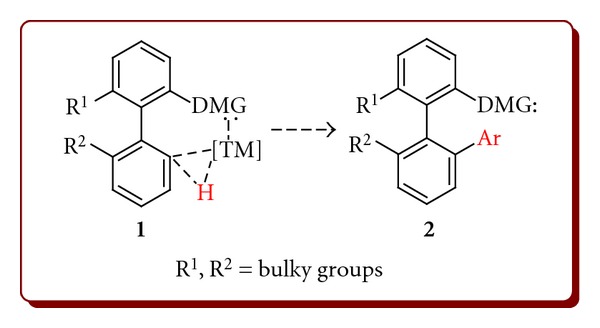
Formation of chiral atropisomeric compounds with the prochiral precursors.

Atropisomeric biaryls constitute an important structural element of many natural products, biologically active compounds, and chiral ligands [1]. Despite significant importance of the synthesis of atropisomeric biaryls (even in the racemic form), their syntheses based on the common cross-coupling reaction such as Suzuki-Miyaura (SM), Negishi, Stille, or Hiyama are still rare what could be explained by easily recognised problems associated with creation of sterically hindered multiply *ortho*-substituted aryl-aryl bonds. Herein, we propose an alternative approach to axially chiral biaryls by selective functionalisation of prochiral substrates, unsubstituted at one or two of the four present *ortho* positions. Thus, introduction of an additional *ortho* substituent into the biaryls already bearing 2 or 3 of them eventually restrains free rotation around the single aryl-aryl bond and creates a pair of atropoisomers (Scheme 1). Such functionalisation could be achieved by classical methods if the position of the functionalisation is unambiguously defined by the substitution pattern, or it could be performed in a catalytic manner, mediated by the transition metal (TM) complex and directed by proper functional groups [2, 3]. Therefore, main impact was made on the synthesis of prochiral precursors of atropisomeric compounds possessing proper functionality for selective introduction of the fourth *ortho* substituent. 

Herein, we would like to present a simple guide for selection of an optimal approach to the synthesis of prochiral biaryls which next could be used in the synthesis of racemic atropisomeric compounds or in their atroposelective synthesis.

## 2. Combinatorial Approach

We have concentrated on the creation of a small library of prochiral biaryls using a combinatorial approach to the SM reactions [4] between several arylboronic acids and arylhalides as well as using other complementary methods. For example, as a result of cross-coupling of nine substrates (both boronic acids and bromoarenes (Table 1)), twenty biaryls could be theoretically obtained.

**Scheme 2:**
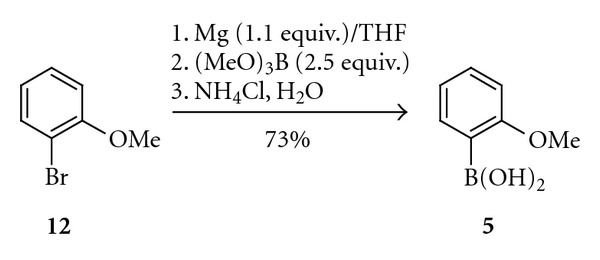
Synthesis of 2-methoxyphenylboronic acid (**5**).

In the case of the preparation of simple tri-*ortho*-substituted biaryls, the syntheses of the individual coupling components could be based on the modified known procedures. The majority of aromatic boronic acids could be obtained by the reaction of organometallic compounds such as Grignard reagents [5], organolithium [6], and organozinc [7] ones with trialkyl borates. Some boronic acid derivatives could also be obtained by the direct C–H activation protocol with utilisation of diboro- and hydridoboro-aromatics [8, 9]. These syntheses are suitable for the production of large quantities of boronic acids in a relatively simple manner. For example, desired 2-methoxyphenylboronic acid (**5**) was obtained by the approach involving the Grignard reagent in 73% isolated yield starting from 2-bromoanisole (**12**)  (Scheme 2). Compounds **3, 4, **and** 6** were prepared in a similar way.

**Scheme 3:**
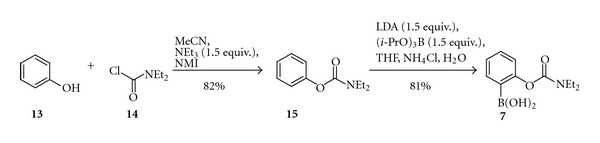
Synthesis of 2-(*N, N-*diethylcarbamyloxy)phenylboronic acid (**7**).

The alternative organolithium approach could be demonstrated by the synthesis of 2-(*N,N-*diethylcarbamyloxy)phenylboronic acid (**7**) obtained by tri-isopropyl borate quench of a suitable organolithium reagent formed in the direct *ortho* lithiation in 81% isolated yield (Scheme 3). According to the standard procedure [6] the solution of *n*-BuLi was added to DIPA solution in THF at −5°C to form LDA. Then, it was chilled down to −76°C, mixed with tri-isopropyl borate and slowly quenched by phenyl diethylcarbamate (**15**). After that the reaction mixture was allowed to warm up to RT and a base was neutralised with the saturated solution of NH_4_Cl, the formed arylboronic acid **7 **was extracted with DCM, dried with MgSO_4_, and eventually purified by flash column chromatography.

**Scheme 4:**
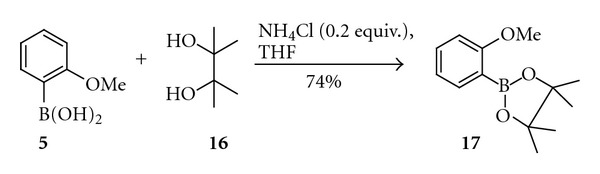
Synthesis of 2-methoxyphenylpinacolborate (**17**).

In the case of difficulties in purification of some boronic acids, they could be easily converted to the corresponding pinacolborates (available from crude boronic acids in a high yielding reaction with pinacol), which usually undergo rapid chromatographic purification [10]. Importantly, the obtained chromatographically pure pinacolborates (e.g., **17**, Scheme 4) could be used in the cross-coupling reaction with the same efficiency as unprotected boronic acids.

**Scheme 5:**
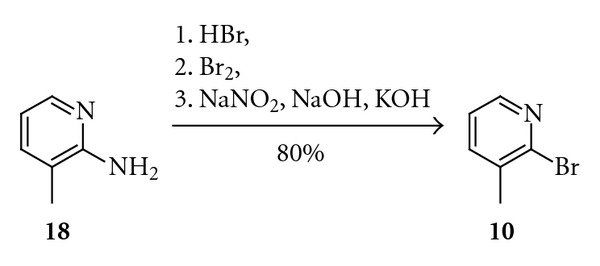
Synthesis of 2-bromo-*β*-picoline (**10**).

Synthesis of aromatic halides or triflates is usually a trivial synthetic task. There is also a large number of these compounds commercially available at reasonable prices. Nevertheless, in some cases, it is more economical to synthesise them by means of one of the many available methodologies. One of the important reactions for obtaining aryl halides is the Sandmeyer reaction [11]. This is a well-known synthesis of aryl halides from aryl diazonium salts. For example, 2-bromo-*β*-picoline (**10**) was rapidly obtained in that reaction from an *in situ* formed heteroaryl diazonium bromide in 80% isolated yield (Scheme 5) [12]. 

**Scheme 6:**
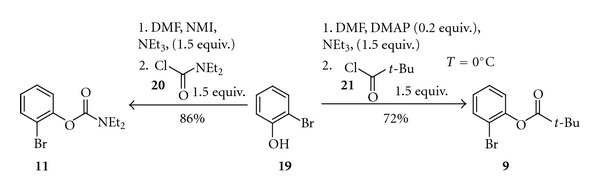
Synthesis of 2-bromophenyl diethylcarbamate (**11**) and 2-bromophenyl pivalate (**9**).

Derivatisation of simple aromatic halides can provide a number of diversified substrates for the cross-coupling reactions. For example, the reactions of 2-bromophenol (**19**) with simple derivatisation agents such as diethylcarbamoyl chloride (**20**) or pivaloyl chloride (**21**) ran in DMF at 0°C and catalysed by *N*-methylimidazole (NMI) or DMAP lead to 2-bromophenyl diethylcarbamate (**11**) and 2-bromophenyl pivalate (**9**) in 86% and 72% yields, respectively (Scheme 6).

Obtained halides **9**, **10**, **11**, and **19** were used as precursors of prochiral biaryls possessing in the *ortho* position to aryl–aryl bond a functional group potentially useful for coordination of transition metals. Therefore, the desired activation of C–H bond and directing effects of the planned transition metal-catalysed reactions at a remote biaryl position as delineated in Scheme 1 could be expected.

## 3. Synthesis of Prochiral Biaryls

### 3.1. Synthesis by the Suzuki Cross-Coupling Reaction

**Scheme 7:**
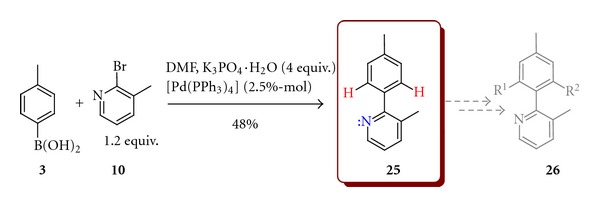
Model synthesis of 3-methyl-2-(4-methylphenyl)pyridine (**25**).

Probably the simplest way to biaryls is via SM coupling of boronic acid with aryl halides. The major limitation of the SM coupling reactions is difficulty in the creation of sterically hindered biaryls possessing more than two *ortho* substituents [13]. At the same time the synthesis of prochiral (doubly or triply *ortho*-substituted biaryls) can be usually performed in high yields. One of the model prochiral biaryls, 3-methyl-2-(4-methylphenyl)pyridine (**25**), was obtained in 48% yield in the reaction of 4-methylphenylboronic acid (**3**) with 2-bromo-*β*-picoline (**10**)  (Scheme 7). This biaryl precursor could be used in the synthesis of chiral atropisomeric compounds **26** in the reaction mediated by TM complexes directed by a lone electron pair of nitrogen and run through the CH activation step.

**Scheme 8:**
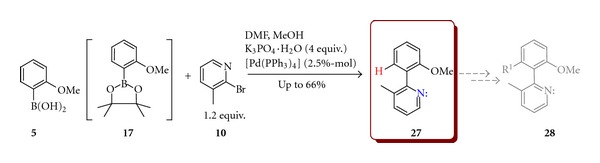
Synthesis of 3-methyl-2-(2′-methoxyphenyl)pyridine (**27**).

The application of pinacoloborates in the SM reaction is well recognised and is frequently utilized in cases where the corresponding boronic acids are difficult to purify. Nevertheless, the direct comparison of efficiency of utilising in coupling reactions boronic acids and the corresponding boranates is very rare. The synthesis of 3-methyl-2-(2′-methoxyphenyl)pyridine (**27**) shown in Scheme 8 provides such an example. The 2-bromo-*β-*picoline (**10**) undergoes the SM reaction with 2-methoxyphenylboronic acid (**5**) or corresponding pinacol ester (**17**) under palladium-catalysed conditions with the yields depending on conditions and substrates used. In order to optimise the reaction conditions several different solvents, bases, catalysts, and additives were tested (Table 2). 

The best yield (Table 2, entry 8) was obtained when 2-methoxyphenylboronic (**5**) acid was utilised in DMF/methanol mixture with tetrakis(triphenylphosphine)palladium(0) as the catalyst. Of many bases used, only potassium phosphate monohydrate secured good yields. The data collected in Table 2 confirmed also a crucial role of water and a strong inorganic base of low nucleophilicity required to achieve reasonable yield in SM reaction. 

As mentioned previously, obtained prochiral biaryl compound **27** could be used in the synthesis of atropisomeric compound **28** by substitution of hydrogen atom in the second *ortho* position with any bulky group (Scheme 8). 

**Scheme 9:**
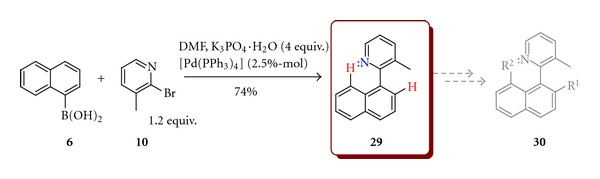
Synthesis 3-methyl-2-(1-naphthyl)pyridine (**29**).

Similarly, prochiral 3-methyl-2-(1-naphthyl)pyridine (**29**) was prepared by coupling of 1-naphtylboronic acid (**6**) with 2-bromo-*β*-picoline (**10**) in 74% yield (Scheme 9) and could be used for the synthesis of atropisomeric compound **30** by substitution of hydrogen in position 2 or 8 of the naphthalene ring directed by a lone electron pair of nitrogen.

**Scheme 10:**
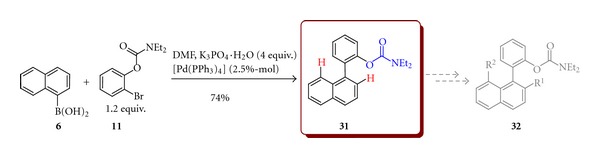
Synthesis of 2-(1-naphthyl)phenyl diethylcarbamate (**31**).

Based on naphthylphenyl core biaryl **31 (**2-(naphthalen-1-yl)phenyl diethylcarbamate), possessing a directing metalation group (DMG), different from nitrogen, was obtained under similar reaction conditions by coupling of 1-naphthylboronic acid (**6**) and 2-bromophenyl diethylcarbamate (**11**) in 74% yield. In this case, diethylcarbamate is the group-directing substitution of hydrogen in the position 2 or 8 of naphthalene ring (Scheme 10).

### 3.2. Synthesis by Functionalisation of Available Substrates

**Scheme 11:**
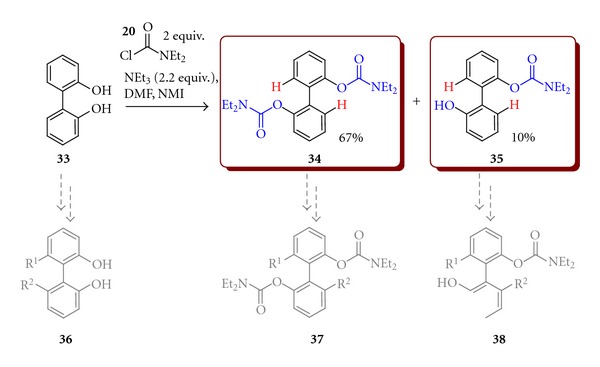
Synthesis of biphenyl-2,2′-diyl bis(diethylcarbamate) (**34**) and 2′-hydroxybiphenyl-2-yl diethylcarbamate (**35**).

**Scheme 12:**
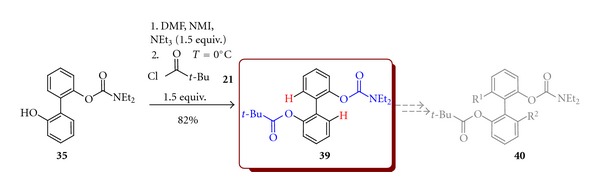
Synthesis of 2′-pivaloyloxy-2′-*N,N-*diethylcarbomoyloxybiphenyl (**39**).

In many cases there is no reason to create biaryls by coupling of two monoaryl compounds because of availability of easy to functionalise biaryls. For example, commercially available 2,2′-biphenol (**33**) could be used as a substrate for synthesis of chiral 3,3′-disubstituted biphenol derivatives. Not only **33** but also its derivatives with the DMG groups such as carbamates could be used for the synthesis of atropisomeric biaryls (Scheme 12). Some DMG substituted derivatives were rapidly prepared from **33**. Thus, the reaction of 2,2′-biphenol with diethylcarbamoyl chloride (**10**) led to biphenyl-2,2′-diyl bis(diethylcarbamate)  (**34**) in 67% yield. 2′-Hydroxybiphenyl-2-yl diethylcarbamate (**35**), which could be used as another prochiral substrate, was also isolated as a side product of this reaction in 10% yield only (Scheme 11).

Mixed *O-*pivaloyl,*O-*carbamoylobiphenol (**39**) was obtained in reaction of **35** with the stoichiometric amount of 2,2-dimethylpropanoyl chloride (**21**) in 82% yield. The obtained product **39 **could be used as a precursor of the synthesis of chiral biaryls in the reaction directed by either pivaloyl or carbamoyl function (Scheme 12). 

### 3.3. Synthesis by the Meyers Reaction

**Scheme 13:**
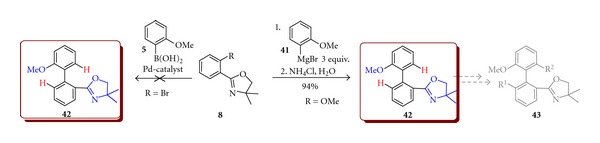
Synthesis of 2-(2′-methoxybiphenyl-2-yl)-4,4-dimethyl-4,5-dihydro-1,3-oxazole (**42**).

A less popular but still powerful method of aryl–aryl bond formation by Meyers reaction [14, 15] could be successfully used when the substitution pattern does not allow to achieve acceptable yield in the TM-mediated cross couplings. For example, for oxazole and oxazoline *ortho*-substituted halogenoarenes as well as boronic acids, the TM catalysed couplings proved to be difficult because of strong interaction of those heterocyclic substituents with the catalysts. Desired oxazole and oxazoline *ortho*-substituted biaryls could be however accessible by the Meyers reaction utilising the *ortho*-methoxy-substituted aryloxazolines (and some other *ortho*-methoxy-substituted aromatics [15]) in good yields (Scheme 13). For example, 2-(2-methoxyphenyl)-4,4-dimethyl-4,5-dihydro-1,3-oxazole (**8**) undergoes the *ipso* nucleophilic substitution reaction when treated with 2-methoxyphenylmagnesium bromide (**41**) and forms prochiral biaryl **42** in 94% yield. Obtained products **42 **may be used for the synthesis of chiral compound **43** by substitution of hydrogen with the bulky group possible in the C–H-activated reaction directed by oxazoline (Scheme 13).

## 4. Application of Prochiral Compounds in the Synthesis of Atropisomeric Biaryls

**Scheme 14:**
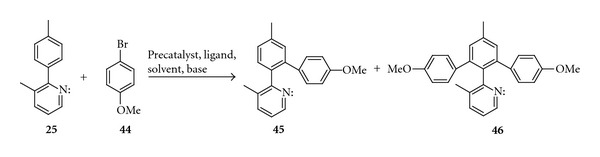
Synthesis of 4-methoxy-5′-methyl-1,1′:2′,1′′-terphenyl (**45**) and 4-methoxy-3′-(4-methoxyphenyl)-5′-methyl-1,1′:2′,1′′-terphenyl (**46**).

To demonstrate a possibility of transformation of prochiral biaryl into the atropisomeric ones, we carried out direct arylation reaction directed by a lone electron pair of nitrogen atom. Thus, the reaction of 3-methyl-2-(p-methylphenyl)pyridine (**25**) with 4-bromoanisole (**44**) led to a mixture of mono- and disubstituted products **45**, and **46 **(Scheme 14).

Obtained monosubstituted product **45**, could be used in the next arylation synthesis with different halogenoarenes (other than 4-MeOC_6_H_4_) to accomplish doubly *ortho* arylated biaryl. High yield in the monosubstitution reaction was obtained when [RuCl_2_(p-cymene)]_2_ was utilised as a catalyst precursor with no phosphorus ligands added (Table 3, entry 7). When tris(pentafluorophenyl)phosphine was used in combination with [RuCl_2_(p-cymene)]_2_ to form a catalyst the formation of disubstituted product **46** in 28% yield was observed (Table 3, entry 6). The most suitable solvent was 1-methyl-2-pyrrolidone (NMP).

The proposed protocol offers an alternative to the traditional aryl–aryl coupling approach to atropisomeric biaryls based on direct and directed by certain DMGs arylation running through the C–H activation reaction step. Of the DMGs promoting aromatic C–H activation reactions, perhaps the most powerful are sp^2^-hybridised nitrogen, carbonyl group, secondary amine, and, rarely, aromatic hydroxyl group. Nevertheless, other functionalities such as nitro group, carbamate, and carboxylate groups as well as those possessing a lone electron pair at a proximal heteroatom, could play a role of a DMG in transition metal mediated C–H activation reactions [16, 17]. The extension of library of DMGs on new functional groups will create an additional opportunity for the synthesis of atropisomeric biaryls. 

## 5. Conclusion

In summary, we have demonstrated a general approach to the synthesis of prochiral biaryls by several complementary methods. The selection of the method was based on the analysis of the availability of starting materials and desirable substitution pattern of the target products. The assumption that easily available prochiral biaryls could be the perspective substrates in the synthesis of atropoismer compounds was confirmed in model direct and directed by the nitrogen lone electron pair transition metal mediated arylation of 2-arylopirydines ran though the CH activation reaction step. The results of the asymmetric direct arylation will be reported separately in due time.

## 6. Experimental

### 6.1. General

All Suzuki coupling reactions were carried out under argon atmosphere using oven-dried glassware and the dry solvent. The reactions were monitored on TLC. The products were purified by distillation or flash column chromatography (Merck silica gel 60 (230–400 mesh)). ^1^HNMR: spectra were recorded on Bruker AVANCE 300 in CDCl_3_; chemical shifts are given in ppm relative to TMS, coupling constants (*J*) in Hz. attenuated total reflection IR spectra were recorded on FTIR Nicolet 8700 A spectrometer and measured in cm^−1^. The HRMS (ESI) measurements were performed on Shimadzu LCMS-IT-TOF instrument. HPLC study was performed on a Merck reversed-phase column: 250 × 4 mm, 5 *μ*m, eluted by methanol/water. All melting points were measured using the Boëtius apparatus and are not corrected. All commercially available substrates were used as received, and all known self-made substrates were examined by comparison with authentic commercial samples.

**4-Methylphenylboronic Acid (4)**

A dried 500 mL flask equipped with a magnetic stirrer, dropping funnel, and reflux condenser was charged with magnesium turnings (1.1 equiv., 80.6 mmol), next, flask was argonated by vacuum/argon triple exchange and a solution of a few crystal of iodine in 10 mL THF was added to activate of the magnesium. Next a solution of 4-bromotoluene (1 equiv., 73 mmol) in dry THF (100 mL) was added dropwise for a period of 1 hour while the reaction mixture was stirred and heated to maintain a gentle reflux. After the additional 1 h refluxing solution was cooled down to −78°C and trimethylborate (22 mL, 183 mmol, 2.5 equiv.) in dry THF (85 mL) was slowly added. The mixture was gradually warmed to room temperature then stirred overnight. Reaction was quenched with saturated aqueous solution of NH_4_Cl (70 mL) and then THF was removed under reduced pressure. The precipitated crystalline **4** was filtered, washed with cold water and next few times with ether diethyl, dried under vacuum. Yield 6.13 g (61%), mp 242–245°C, (Lit.[18] 256–263°C).

**4-Methoxyphenylboronic acid (3) was prepared in a similar way as 4**

Yield 17.9 g, 56%, mp 202°C, (Lit. [19] 202–204°C).

**2-Methoxyphenylboronic acid (5) was prepared in a similar way as 4**

Yield 17.6 g, 73%, mp 99°C, (Lit. [20] 105°C).

**1-Naphtylboronic acid (6) was prepared in a similar way as 4**

Yield 21.1 g, 85%, mp 217–219°C, (Lit. [21] 202–203°C).

**2-(*N,N-*diethylcarbamoyloxy)phenylboronic acid (7) was obtained in two steps**

(1) A 100 mL flask equipped with a magnetic stirrer was charged with phenol (53.2 mmol, 1 equiv.), 50 mL acetonitrile, 4.5 mL *N*-methylimidazole, triethylamine (79.8 mmol, 1.5 equiv.), and diethylcarbamoyl chloride (**14**)  (79.8 mmol, 1.5 equiv.). The flask was closed with a tight PTFE stopper and heated at 100°C for 24 h. After that time, the reaction solution was cooled down to room temperature and the solvents were removed under reduced pressure. 100 mL water was added to a residue, and product was extracted with diethyl ether (90 mL), washed with water (80 mL) and 5% aq. NaOH (30 mL). The organic phase was separated and dried with MgSO_4_. Solvent was evaporated in vacuum and the remaining residue was distilled under the reduced pressure of 1 mmHg, the product was collected in fraction at about 100°C. Yield 8.4 g (82% yield). ^1^H NMR (300.33 MHz, CDCl_3_): *δ* = 1.23–1.27 (m, 6H) 3.40–3.47 (m, 4H) 7.12–7.23 (m, 3H) 7.34–7.40 (m, 2H). (Lit. [22]).
(2) A 100 mL flask equipped with a magnetic stirrer was argonated by vacuum/argon triple exchange and charged with DIPA (10.2 mmol, 1.5 equiv.) and 30 mL THF next cooled down to −30°C followed by 1.6 M solution of* n*-BuLi in hexane (10.2 mmol, 1.5 equiv.) was slowly added. The LDA solution was stirred for 30 min at −30°C and then cooled down to −78°C. A solution of tri-isopropyl borate (10.2 mmol, 1.5 equiv.) in 10 mL THF was injected to the reaction mixture followed by a solution of **15** (6.8 mmol, 1 equiv.) in 10 mL THF was slowly added. All the time, the reaction temperature was maintain between −78°C and −70°C. After addition, the reaction mixture was allowed to warm to room temperature and was quenched with saturated aqueous solution of NH_4_Cl (60 mL). THF was removed under reduced pressure, and the product was extracted from residue with dichloromethane (60 mL). The organic phase was removed, washed with diluted aq. NaHCO_3_ (3 × 20 mL), next with water (2 × 20 mL) and dried with MgSO_4_. Solvent was evaporated in vacuum, and the remaining pure product **7** was collected. Yield 3.96 g (81%, mp 162–165°C). ^1^H NMR (300.33 MHz, CDCl_3_): *δ* = 1.04–1.13 (m, 6H) 3.22–3.32 (m, 4H) 7.02 (d, *J* = 8.05, 1H) 7.18–7.23 (m, 1H) 7.33–7.39 (m, 1H) 7.85–7.88 (m, 1H), ^13^C NMR (62.90 MHz, CDCl_3_): *δ* = 11.45, 12.03, 40.09, 40.52, 74.84, 75.34, 75.86, 117.54, 123.20, 128.44, 133.60, 153.62 (Lit. [23]).

**2-Methoxyphenylpinacolborate (17)**

A 50 mL flask equipped with a magnetic stirrer was charged with 2-methoxyphenylboronic acid** (5) **(13.1 mmol, 1 equiv.), 30 mL of THF, pinacol (**16**)  (15.8 mmol, 1.2 equiv.), and NH_4_Cl (2.6 mmol, 0.2 equiv.) then heated at 40°C for 24 hours. After that time, THF was removed under reduced pressure and product was crystallised from petroleum ether. Yield 2.31 g (73%), mp 80–82°C (Lit. [10, 24] 80–81°C).

**2-Bromo-*β*-picoline (10)**

A 100 mL flask equipped with a magnetic stirrer and thermometer was charged with 40% aqueous solution of HBr (39.5 mL), cooled down to −10°C, and 2-amino-*β*-picoline(**18**)  (80 mmol) was slowly added. The temperature was kept below 0°C while Br_2_ (0.23 mmol) was added over a period of 2 h. After that, a solution of NaNO_2_ (0.2 mol) in H_2_O (20 mL) was slowly added at the same temperature, and the mixture was stirred for next 30 minute. The mixture was gradually warmed to room temperature and was stirred 1 hour more. After that solution of NaOH (0.75 mol) in H_2_O (30 mL) and solid KOH (90 mmol) were added. After 1 hour of stirring products were extracted with diethyl ether. The organic phase was separated and dried with MgSO_4_. Solvent was evaporated in vacuum and the remaining residue was distilled to give 8.16 g (80%) of **10.** Bp 69°C at 12 mmHg (Lit. [8] 76-77°C at 7 mmHg).

**2-Bromophenyl Pivalate (9)**

A 100 mL flask equipped with a magnetic stirrer charged with 2-bromophenol (**19**)  (11.6 mmol, 1 equiv.), 20 mL dichloromethane, 1.1 mL *N-*methylimidazole, and triethylamine (1.76 g, 17.4 mmol, 1.5 equiv.) was cooled down to 0°C, and pivaloyl chloride (**21**)  (17.4 mmol, 1.5 equiv.) was added. The mixture next was stirred for 2 hours at room temperature, the solvents were removed under reduced pressure, and a residue was diluted with water. The product was extracted with diethyl ether and washed with aqueous 1 M HCl, saturated solution of NaHCO_3_ and water again. The organic phase was separated and dried with MgSO_4_. Solvent was evaporated in vacuum and the remaining residue was distilled to give 2.73 g (90%) of **9**. Bp 115°C at 1 mmHg. ^1^H NMR (300.33 MHz, CDCl_3_): *δ* = 1.43 (sc, 9H) 7.10–7.15 (m, 2H) 7.28–7.37 (m, 1H) 7.60–7.63 (m, 1H). (Lit [25]).

**2-Bromophenyl diethylcarbamate (11) was prepared in a similar way as 9, but DMF was used as a solvent instead of DCM**

Yield 1.26 g (86%), bp 125°C at 1 mmHg. (Lit. [26]).

**3-Methyl-2-(4-methylphenyl)pyridine (25)**

A 50 mL flask equipped with a magnetic stirrer was argonated by vacuum/argon triple exchange and charged with 4-methoxyphenylboronic acid (**3**)  (21.8 mmol, 2 equiv.), 2-bromo-*β*-picoline (**10**)  (10.9 mmol, 1 equiv.), K_3_PO_4_·H_2_O (43.6 mmol), 30 mL DMF, and [Pd(PPh_3_)_4_] (0.27 mmol). The flask was closed with a tight PTFE stopper and heated at 130°C for 24 h. The reaction mixture was allowed to cool down to room temperature and DMF was removed under reduced pressure. The product was extracted with diethyl ether and washed with water. The organic phase was separated and dried with MgSO_4_. Solvent was evaporated in vacuum and the remaining residue was purified by column chromatography on silica gel using hexane/isopropanol (9/1) as an eluent. Yield 1 g (48%). ^1^H NMR (300.33 MHz, CDCl_3_): *δ* = 2.38 (sc, 3H) 2.42 (sc, 3H) 7.16 (q, *J* = 4.76, 2.93 Hz, *J* = 4.76 Hz, 1H) 7.45 (d, *J* = 8.05 Hz, 2H) 7.28 (d, *J* = 7.68 Hz, 2H) 7.58 (d, *J* = 7.50 Hz, 1H) 8.54 (d, *J* = 4.76 Hz, 1H). (Lit. [27]).

**3-Methyl-2-(2-methoxyphenyl)pyridine (27) was prepared in a similar way as 25. Yield 435 mg (66%)**

^1^H NMR (300.33 MHz, CDCl_3_): *δ* = 2.18 (sc, 3H) 3.78 (sc, 3H) 6.98 (d, *J = *8, 2 Hz, 1H) 7.06 (t, *J* = 7.5, 8.5 Hz, 1H) 7.20 (q, *J* = 4.7, 2.9, 4.8 Hz, 1H) 7.29 (dd, *J* = 1.8, 4.6, 1.8 Hz, 1H) 7.36–7.42 (m, 1H) 7.57 (d, *J* = 8.6 Hz, 1H) 8.53 (d, *J* = 5.8 Hz, 1H); ^13^C NMR (62.90 MHz, CDCl_3_): *δ* = 19.39, 55.87, 111.24, 121.20, 122.61, 125.30, 129.91, 130.93, 137.8, 145.94, 147.02 (Lit. [28]).

**2-(1-Naphthyl)phenyl diethylcarbamate (29) was prepared in a similar way as 25**

Yield 483 mg (74%); ^1^H NMR (300.33 MHz, CDCl_3_): *δ* = 0.37–1.36 (m, 6H) 2.62–3.54 (m, 4H) 7.07–7.66 (m, 9H) 7.85–7.89 (m, 2H); ^13^C NMR (62.90 MHz, CDCl_3_): *δ* = 13.42, 41.41, 122.19, 123.45, 125.65, 126.05, 126.32, 126.77, 127.03, 127.83, 128.18, 128.34, 129.07, 129.61, 131.92, 133.54, 148.27, 151.64, 159.83; IR (neat) *ν* = 3050, 3000, 1992, 1892, 1832, 1587, 1568, 1505, 1468, 1433, 1388, 1254, 1218, 1197, 1178, 1133, 1108, 1016, 971, 918, 873,788, 685, 657, 626, 572, 550, 443; HRMS (ESI): m/z = 220.1125 [C_16_H_13_N+H]^+^, m/z (teor.) = 220.1121, diff. = 1.82 ppm.

**3-Methyl-2-(1-naphthyl)pyridine (31) was prepared in similar way as 25**

Yield 781 mg (74%); ^1^H NMR (300.33 MHz, CDCl_3_): *δ* = 2.11 (sc, 3H) 7.30 (dd, *J* = 4.94, 2.93, 4.94 Hz, 1H) 7.41–7.68 (m, 4H) 7.59 (t, *J* = 6.95, 8.23 Hz, 1H) 7.67 (d, *J* = 7.68 Hz, 1H) 7.93 (d, *J* = 8.23 Hz, 2H) 8.64 (d, *J* = 4.39 Hz, 1H); ^ 13^C NMR (62.90 MHz, CDCl_3_): *δ* = 19.72, 122.96, 125.79, 125.85, 126.25, 126.72, 126.82, 128.73, 128.78, 131.85, 131.98, 132.98, 134.14, 138.30, 147.37, 158.96; IR (neat) *ν* = 3058, 2973, 2933, 2874, 1712, 1592, 1509, 1471, 1457, 1414, 1379, 1316, 1272, 1259, 1200, 1151, 1096, 1045, 956, 937, 802, 778, 751, 618. HRMS (ESI): m/z = 320.1630 [C_21_H_21_NO_2_+H]^+^, m/z (teor.) = 320.1645, diff. = 4.69 ppm.

**Biphenyl-2,2′-diyl bis(diethylcarbamate) (34)**

A 100 mL flask equipped with a magnetic stirrer was charged with 2,2′-biphenol (**33**)  (26.8 mmol, 1 equiv.), dimethylformamide (30 mL), *N*-methylimidazole (2.5 mL), triethylamine (5.4 g, 53.7 mmol, 2.2 equiv.) and diethylcarbamoyl chloride (**20**)  (7.3 g, 2.2 equiv.). The flask was closed with a tight PTFE stopper and heated at 100°C for 24 h. After that time the reaction solution was cooled down to room temperature and the solvents were removed under reduced pressure. 100 mL water was added to a residue and the product was extracted with diethyl ether (90 mL), washed with water (80 mL), and 5% aq. NaOH (30 mL). The organic phase was separated and dried with MgSO_4_. Solvent was evaporated in vacuum and the remaining residue was purified by column chromatography on silica gel using hexane/acetone (9/1) as an eluent. Yield 6.87 g (67%). ^1^H NMR (300.33 MHz, CDCl_3_): *δ* = 0.86–1.03 (m, 12H) 3.11–3.21 (m, 8H) 7.18–7.38 (m, 8H). (Lit. [29]).

**2′-hydroxybiphenyl-2-yl diethylcarbamate (35) was also obtained in this reaction leading to 34 in 0.79 g (10%) yield**

^1^H NMR (300.33 MHz, CDCl_3_): *δ* = 0.92–1.04 (m, 6H) 3.18–3.23 (m, 4H) 6.92–6.95 (m, 1H) 6.98–7.01 (m, 1H) 7.12 (dd, *J* = 2.20, 6.22, 1.28, 1H), 7.22–7.29 (m, 2H) 7.33 (d, *J* = 3.48 Hz, 1H) 7.35 (sc, 1H) 7.42–7.47 (m, 1H); IR (neat) *ν* = 3260, 3061, 2976, 2935, 2875, 2707, 1683, 1606, 1573, 1506, 1483, 1442, 1426, 1379, 1364, 1275, 1197, 1165, 1118, 1095, 1047, 1006, 966, 938, 842, 818, 748, 618, 574, 496, 432; HRMS (ESI): m/z = 308.1240 [C_17_H_19_NO_3_+Na]^+^, m/z (teor.) = 308.1257, diff. = 5.52 ppm.

**2-Pivaloyloxy-2′-*N,N-*diethylcarbomoyloxybiphenyl (39)**

A 100 mL flask equipped with a magnetic stirrer charged with 0.3 g 2′-hydroxybiphenyl-2-yl diethylcarbamate (**35**)  (1 mmol, 1 equiv.), 1.8 mL dimethylformamide, 0.1 mL *N*-methylimidazole, and 0.2 mL triethylamine (5.4 g, 53.7 mmol, 2.2 equiv.) was cooled down to 0°C, and pivaloyl chloride (**21**)  (126.7 mg, 1 mmol, 1 equiv.) was added. The content were stirred for 2 h at 0°C. The mixture next was stirred for 2 hours at room temperature; the solvents were removed under reduced pressure and a residue was diluted with water. The product was extracted with diethyl ether and washed with aqueous 1 M HCl. The organic phase was separated and dried with MgSO_4_. Solvent was evaporated in vacuum, and the remaining residue was purified by column chromatography on silica gel using hexane/acetone (99/1) as an eluent. Yield 318 mg (82%). ^1^H NMR (300.33 MHz, CDCl_3_): *δ* = 0.87–1.03 (m, 15H) 3.12–3.22 (m, 4H) 7.08–7.11 (m, 1H) 7.19–7.40 (m, 7H). IR (neat) *ν* = 2973, 2933, 2874, 1749, 1715, 1471, 1414, 1380, 1367, 1270, 1250, 1227, 1198, 1153, 1110, 1045, 1010, 959, 938, 897, 749. HRMS (ESI): m/z = 392.1855 [C_22_H_27_NO_4_+Na]^+^, m/z (teor.) = 392.1832, diff. = 5.86 ppm. (Lit. [30]).

**2-(2′-Methoxybiphenyl-2-yl)-4,4-dimethyl-4,5-dihydro-1,3-oxazole (42)**

A dried Schlenk equipped with a magnetic stirrer was argonated by vacuum/argon triple exchange and charged with the 1 M THF solution of Grignard reagent **41** (3.4 mmol, 3 equiv.) then 2-(2-methoxyphenyl)-4,4-dimethyl-4,5-dihydro-1,3-oxazole (**8**)  (1.1 mmol, 1 equiv.) was added and the reaction mixture was heated overnight at 80°C. The reaction was allowed to cool down to room temperature and it was quenched with saturated aqueous solution of NH_4_Cl (3 mL). The solvents were removed under reduced pressure, 100 mL of water was added to a residue and products were extracted with diethyl ether. The organic phase was separated and dried with MgSO_4_. Solvent was evaporated in vacuum and the remaining residue was purified by column chromatography on silica gel using hexane/acetone (99/1) as an eluent. Yield 299 mg (94%) of product. ^1^H NMR (300.33 MHz, CDCl_3_): *δ* = 1.27 (sc, 6H), 3.75 (sc, 3H), 3.78 (sc, 2H), 6.89–6.92 (m, 1H), 7.02 (t, *J* = 7.50, 7.50 Hz, 1H), 7.24–7.41 (m, 4H), 7.47–7.52 (m, 1H), 7.85–7.88 (m, 1H). (Lit. [31]).

**4-Methoxy-5′-methyl-1,1′:2′,1′′-terphenyl (45), 4-methoxy-3′-(4-methoxyphenyl)-5′-methyl-1,1′:2′,1′′-terphenyl (46)**

A 50 mL flask equipped with a magnetic stirrer was argonated by vacuum/argon triple exchange and charged with [RhCl(cod)]_2_ (3 *μ*mol, 2.5 mol%), tris(pentafluorophenyl)phosphine (2%-mol), and 2 mL *N*-methylpyrrolidone (NMP). The reaction mixture stirred for 30 minute at 70°C, cooled down to room temperature and to solution of formed catalyst 3-methyl-2-(4-methylphenyl)pyridine (**25**)  (1.4 mmol, 1 equiv.), *p*-bromoanisole (**44**)  (2.8 mmol, 2 equiv.), Cs_2_CO_3_ (2.7 mmol, 2 equiv.) and 1 mL NMP were added. The solution was heated at 160°C for 24 h, after that the reaction mixture was cooled down to room temperature. The products were extracted with diethyl ether and washed with water. The organic phase was separated and dried with MgSO_4_. Solvents were evaporated in vacuum, and the remaining residue was purified by column chromatography on silica gel using hexane/acetone (6/1) as an eluent to afford **45** (290 mg, 64%) and **46 **(trace).

**45:** ^1^H NMR (300.33 MHz, CDCl_3_): *δ* = 2.47 (sc, 3H), 3.74 (sc, 6H), 6.66–6.69 (m, 4H), 6.89–7.28 (m, 9H), 8.29 (d, *J* = 4.76, 1H). IR (neat) *ν* = 3002, 2953, 2923, 2858, 2835, 1608, 1581, 1568, 1515, 1496, 1460 1441, 1423, 1380, 1290, 1245, 1177, 1137, 1111, 1066, 1051, 1031, 989, 827, 788, 597, 566, 520. HRMS (ESI): m/z = 290.1535 [C_20_H_19_NO+H]^+^, m/z (teor.) = 290.1539, diff. = 1.38 ppm.
**46:** ^1^H NMR (300.33 MHz, CDCl_3_): *δ* = 1.76 (sc, 3H) 2.46 (sc, 3H) 3.76 (sc, 3H) 6.71 (d, *J* = 8.78, 2H) 7.04 (d, *J* = 8.78, 2H) 7.08–7.12 (m, 1H) 7.22–7.32 (m, 5H) 8.50–8.52 (m, 1H). IR (neat) *ν* = 2957, 2928, 2835, 1710, 1607, 1576, 1510, 1460, 1441, 1421, 1401, 1379, 1290, 1243, 1176, 1109, 1031, 830, 793, 731, 595, 568, 548, 530. HRMS (ESI): m/z = 396.1958 [C_27_H_25_NO_2_+H]^+^, m/z (teor.) = 396.1958, diff. = 0.00 ppm.

## Figures and Tables

**Table 1:** Aromatic boronic acids and bromoaryls for the cross-coupling reaction.

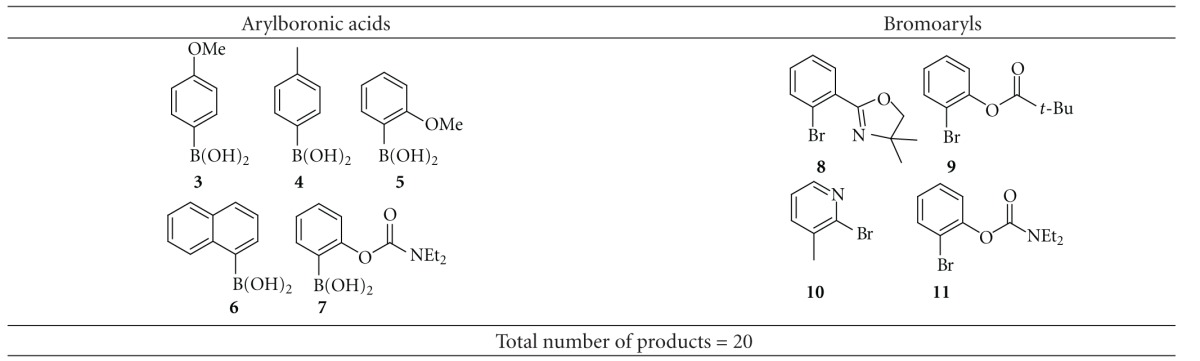

**Table 2:** Optimisation of the reaction conditions of synthesis of 3-methyl-2-(2′-methoxyphenyl)pyridine (**27**).

Entry	Substrate	Solvent	Base	Catalyst	Yield [%]^a^
1	**17**	Toluene/ethanol	Na_2_CO_4_ (aq.)	[Pd(PPh_3_)_4_]	trace
2	**5**	DMF (anh.)	K_3_PO_4_ (anh.)	[Pd(PPh_3_)_4_]	46
3	**5**	DMF (anh.)	K_3_PO_4_·H_2_O	[Pd(PPh_3_)_4_]	49
4	**5**	DMF	K_3_PO_4_·H_2_O	[Pd(PPh_3_)_4_]	57
5	**5**	DMF	K_3_PO_4_·H_2_O	Ph_3_P, Pd (AcO)_2_	54
6	**5**	MeOH	K_3_PO_4_·H_2_O	[Pd(PPh_3_)_4_]	42^b^
7	**17**	MeOH	K_3_PO_4_·H_2_O	[Pd(PPh_3_)_4_]	59
8	**5**	DMF/MeOH	K_3_PO_4_·H_2_O	[Pd(PPh_3_)_4_]	66
9	**5**	MeOH/H_2_O	AgOAc	[Pd(PPh_3_)_4_]	0
10	**5**	MeOH/H_2_O	Ag_2_CO_3_	[Pd(PPh_3_)_4_]	0

^a^Reactions were carried out under argon atmosphere, under gentle reflux. ^b^Reaction run at 130°C in sealed vessel.

**Table 3:** Optimisation of direct arylation reaction.

Entry	Base	Catalyst	Ligand	**45** [%]	**46** [%]	Conversion [%]
1	Cs_2_CO_3_	[RhCl(cod)]_2_	none	2	3	5
	Ag_2_CO_3_					
2	Cs_2_CO_3_	[RhCl(cod)]_2_	none	32 (36)^b^	2	34
3	Cs_2_CO_3_	[RhCl(cod)]_2_	PPh_3_	4^a^	3^a^	7^a^
4	Cs_2_CO_3_	[RhCl(cod)]_2_	*S-*Phos	72 (64)^b^	4	76
5	Cs_2_CO_3_	[RuCl_2_(p-cymene)]_2_	PPh_3_	1^a^	1^a^	2^a^
6	Cs_2_CO_3_	[RuCl_2_(p-cymene)]_2_	tris(pentafluorophenyl)-phosphine	28	31	59
7	Cs_2_CO_3_	[RuCl_2_(p-cymene)]_2_	none	71	13	84
8	Cs_2_CO_3_	[RuCl_2_(p-cymene)]_2_	tris(2,4,6-trimethoxy-phenyl)phosphine	24	18	42
9	Cs_2_CO_3_	[RuCl_2_(p-cymene)]_2_	dppe	29	7	36
10	K_2_CO_3_	[RuCl_2_(p-cymene)]_2_	*S-*Phos	54	33	87

The reactions were carried out under argon atmosphere, in NMP, at 160°C, for 24 hours, HPLC yields, ^a^2 equiv. of compound **44** were used in the reaction. ^b^The isolated yields are given in the parentheses.
